# Focal dynamic thermal imaging for label-free high-resolution characterization of materials and tissue heterogeneity

**DOI:** 10.1038/s41598-020-69362-8

**Published:** 2020-07-28

**Authors:** Christine M. O’Brien, Hongyu Meng, Leonid Shmuylovich, Julia Carpenter, Praneeth Gogineni, Haini Zhang, Kevin Bishop, Suman B. Mondal, Gail P. Sudlow, Cheryl Bethea, Clyde Bethea, Samuel Achilefu

**Affiliations:** 1 Department of Radiology, Washington University School of Medicine, 4515 McKinley Ave., Couch Biomedical Research Building, St. Louis, MO 63110 USA; 2 Quantum Technology Consultants, Inc., 8 Grist Mill Lane, Franklin Park, NJ 08823 USA; 3 Department of Biomedical Engineering, Washington University in St. Louis, St. Louis, MO 63130 USA; 4 Department of Medicine, Washington University School of Medicine, St. Louis, MO 63110 USA; 5 Department of Biochemistry and Molecular Biophysics, Washington University School of Medicine, 660 South Euclid Avenue, St. Louis, MO 63110 USA

**Keywords:** Engineering, Biomedical engineering, Imaging, Optical imaging

## Abstract

Evolution from static to dynamic label-free thermal imaging has improved bulk tissue characterization, but fails to capture subtle thermal properties in heterogeneous systems. Here, we report a label-free, high speed, and high-resolution platform technology, focal dynamic thermal imaging (FDTI), for delineating material patterns and tissue heterogeneity. Stimulation of focal regions of thermally responsive systems with a narrow beam, low power, and low cost 405 nm laser perturbs the thermal equilibrium. Capturing the dynamic response of 3D printed phantoms, ex vivo biological tissue, and in vivo mouse and rat models of cancer with a thermal camera reveals material heterogeneity and delineates diseased from healthy tissue. The intuitive and non-contact FDTI method allows for rapid interrogation of suspicious lesions and longitudinal changes in tissue heterogeneity with high-resolution and large field of view. Portable FDTI holds promise as a clinical tool for capturing subtle differences in heterogeneity between malignant, benign, and inflamed tissue.

## Introduction

Applications of thermal imaging in materials science, security, and medicine have surged in recent years, due in part to advances in infrared sensor technology^1–3^. Relying on the emissive properties of living systems, thermal imaging has been used to generate heat maps of individuals and detect inflammation. Despite its advantages of being a handheld, real-time, and non-contact measurement technique, the poor specificity of thermal imaging has prevented widespread adoption in the clinic^4^. To overcome this limitation, techniques that probe thermal tissue properties, rather than equilibrium temperature, have been developed. These dynamic thermal imaging (DTI) techniques apply a hot or cold thermal stimulus to tissue and observe its rate of recovery. Leveraging the different rates of thermal recovery in healthy versus diseased tissue following the application of thermal stimulus^5^, DTI has demonstrated improved tumor detection accuracy^5–8^. However, label-free DTI fails to fully capture high-resolution thermal tissue heterogeneity, which can highlight subtle differences for distinguishing malignant, benign, or inflamed tissue^9,10^. This resolution limit is due to how a thermal stimulus is applied. In existing implementations of DTI, a thermal stimulus is applied over a large area of tissue, which blurs the detection of unique thermal properties of small areas within the thermally perturbed region. Super-resolution DTI rasters focal excitation of visible light across the sample, followed by image reconstruction that determines the centroid of absorptive agents well beyond the diffraction limit to improve spatial resolution^11^. For biological samples, exogenous contrast agents such as dyes or nanoparticles are used to generate sufficient contrast. While this technique is an exciting advancement for microscopy applications, in vivo or point-of-care applications would be complicated by long imaging time (minutes rather than seconds), complex system setup, and reliance upon exogenous contrast agents. While exogenous agents are particularly useful in an ex-vivo setting, for in-vivo applications, they add regulatory hurdles for product approval that may limit their clinical applicability. As such, a gap exists between low-resolution but high field of view wide-field DTI that has seen multiple in vivo applications, and super-resolution DTI, which holds great promise ex vivo.

**Figure 1:**
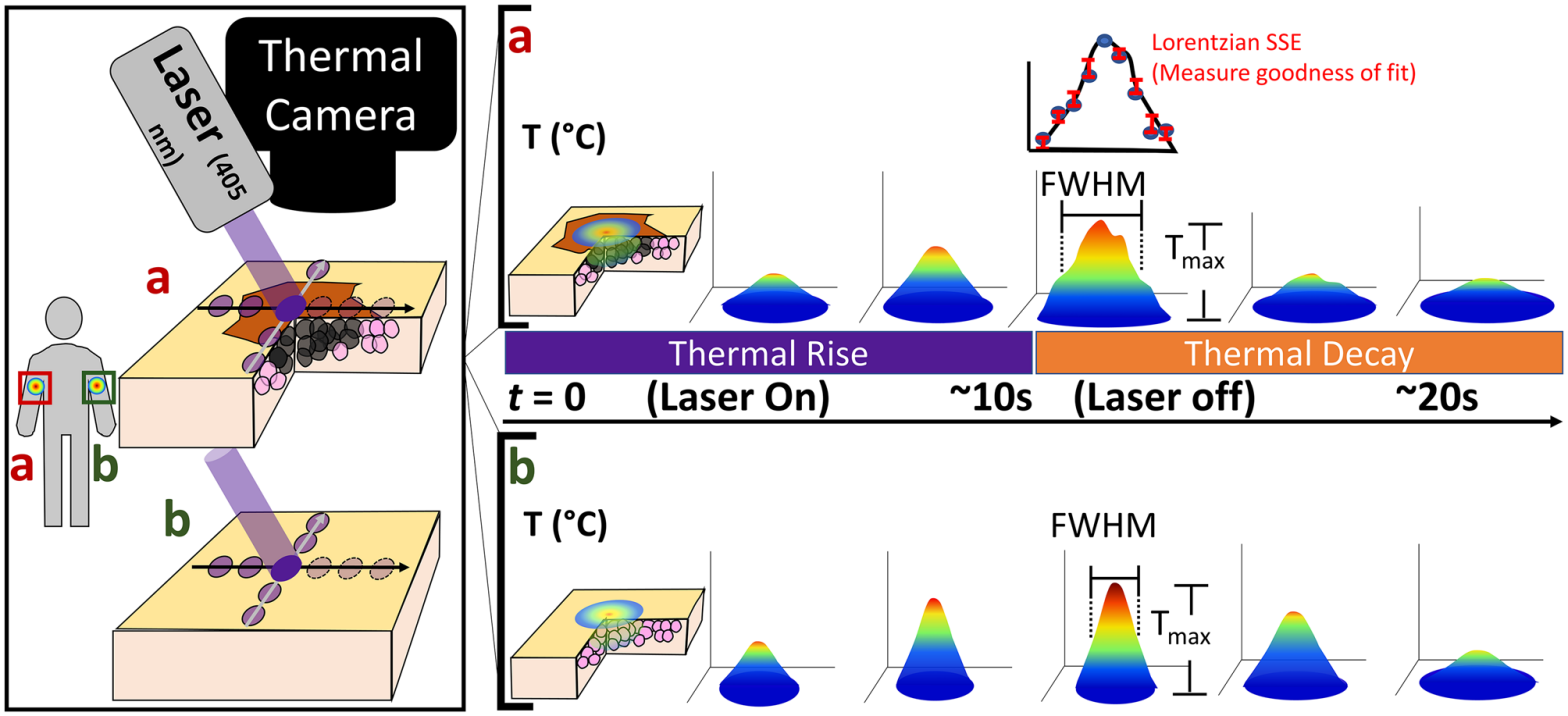
Focal dynamic thermal imaging phenomenon and features used for analysis. FDTI consists of a sequence of light absorption, heat generation, thermal perturbation, and thermal recovery processes. FDTI uses a small diameter laser beam to irradiate an area of interest (**a**) as well as an area serving as a healthy control site (**b**). Optical tissue properties determine the degree and distribution of local heating due to laser irradiation. Once the laser irradiation is complete, the thermal decay phase begins. The surface thermal profile, which is measured through both thermal rise and decay phases with a thermal camera, can be visualized as a 3D plot defined by multiple features including the maximum temperature (Tmax) and full width at half-maximum (FWHM). These features can be analyzed over both thermal rise and thermal decay phases. Quantitative analysis of thermal profile features in different tissue types reports underlying tissue heterogeneity.

Here, we report the development of a new label-free dynamic photothermal imaging technique, focal dynamic thermal imaging (FDTI), which overcomes the spatial limitations of current DTI strategies while using simple hardware and analysis methods compared to super resolution DTI. Abundance of light-absorbing chromophores creates heat upon exposure to radiation. Leveraging this phenomenon, we postulated that the skin would efficiently absorb a narrow beam of 405 nm light to generate a focal thermal source that propagates radially from the heat source through the tissue along a temperature gradient. The use of focused 405 nm light allows for thermal contrast generation through endogenous chromophores rather than exogenous dyes. However, care must be taken to ensure laser irradiation does exceed doses that may be associated with DNA damage^12^. A thermal camera detects the ensuing perturbation and recovery of the thermal equilibrium. While tissue-specific optical properties define the degree and distribution of light absorption and subsequent heat generation, tissue properties such as thermal conductivity, specific heat, density, perfusion, metabolic rate, and the baseline temperature dictate heat propagation (Fig. 1). Assessment of this new technique using custom 3D printed thermal resolution targets showed higher spatial resolution and contrast than conventional DTI. In vivo, FDTI was able to distinguish healthy and cancerous tissue in animal models of cancer with over 90% accuracy. The label-free, non-contact, intuitive, and portable FDTI system provides high-resolution and high contrast images in real-time for use in diverse applications, including materials characterization and disease diagnosis.

## Results

### Computational modeling

**Figure 2:**
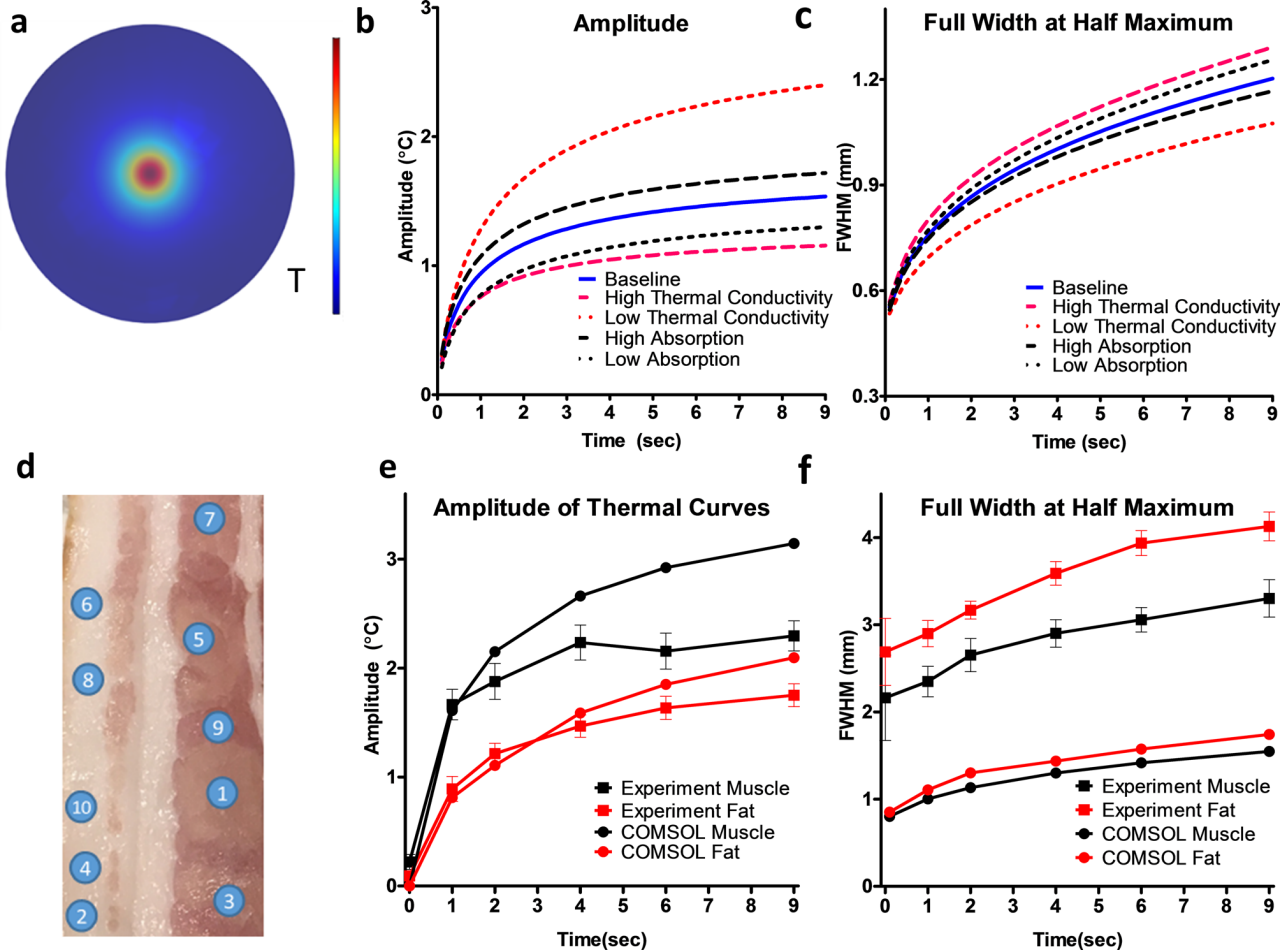
FDTI simulation and validation. (**a**) COMSOL simulation demonstrating radial heating from laser stimulation during the heat rise phase of FDTI; (**b**) amplitude and (**c**) FWHM results from simulated parameter sweep; (**d**) porcine tissue measured for experimental validation of COMSOL model; (**e**) amplitude and (**f**) FHWM comparison between simulated and experimentally measured values of porcine muscle and fat tissue.

A 3D model of the FDTI phenomenon was developed in COMSOL to demonstrate the feasibility of using this technique to measure unique signals from different materials and tissue types. The impact of each variable specified in the Pennes’ bioheat equation^13^ on heat generation and transfer was evaluated using a parameter sweep across high and low biological values. The simulations were analyzed by plotting cross-sections of the thermal peak along the diameter of the laser spot and extracting the amplitude and full width at half maximum (FWHM). Simulations depicted in Fig. 2a illustrate the expected radial heat propagation away from the initial thermal source initiated by light absorption. As expected, the parameter sweep of thermal and optical properties show that thermal conductivity and optical absorption coefficient greatly affect the resulting FDTI signals (Fig. 2b, c).

The model was validated experimentally using porcine tissue with well-differentiated muscle and fat compartments (Fig. 2d–f) using the same laser power and irradiation duration in the model. The FWHM and amplitude of the thermal peak revealed similar trends in the experiment and COMSOL simulation results, verifying that the trends observed in the parameter sweep reflect results from biological experiments. The COMSOL model provides a numerical basis for using FDTI to detect distinct thermal characteristics in different tissue types.

### Data acquisition and analysis

FDTI data were acquired with a thermal camera. The images can be visualized as 3D plots with spatial location in x and y, and temperature in z at a fixed time *t*. Prior to laser stimulation, the 3D plot is essentially flat and reflects steady state skin temperature. A thermal peak grows exponentially with time in the area of laser stimulation. After turning off the laser, the heat decays exponentially as a function of tissue thermal properties. Common features extracted from FDTI, either from single frames or throughout heating, include thermal peak amplitude, width, FWHM, area, and volume. Furthermore, exponential growth and exponential decay constants can be fit to the maximum temperature over time (Fig. 1). Additionally, in a perfectly homogeneous sample, the thermal peak is smooth and radially symmetric. Small scale heterogeneity in tissue thermal properties perturbs the expected smooth and radially symmetric image.

### FDTI spatial resolution

**Figure 3:**
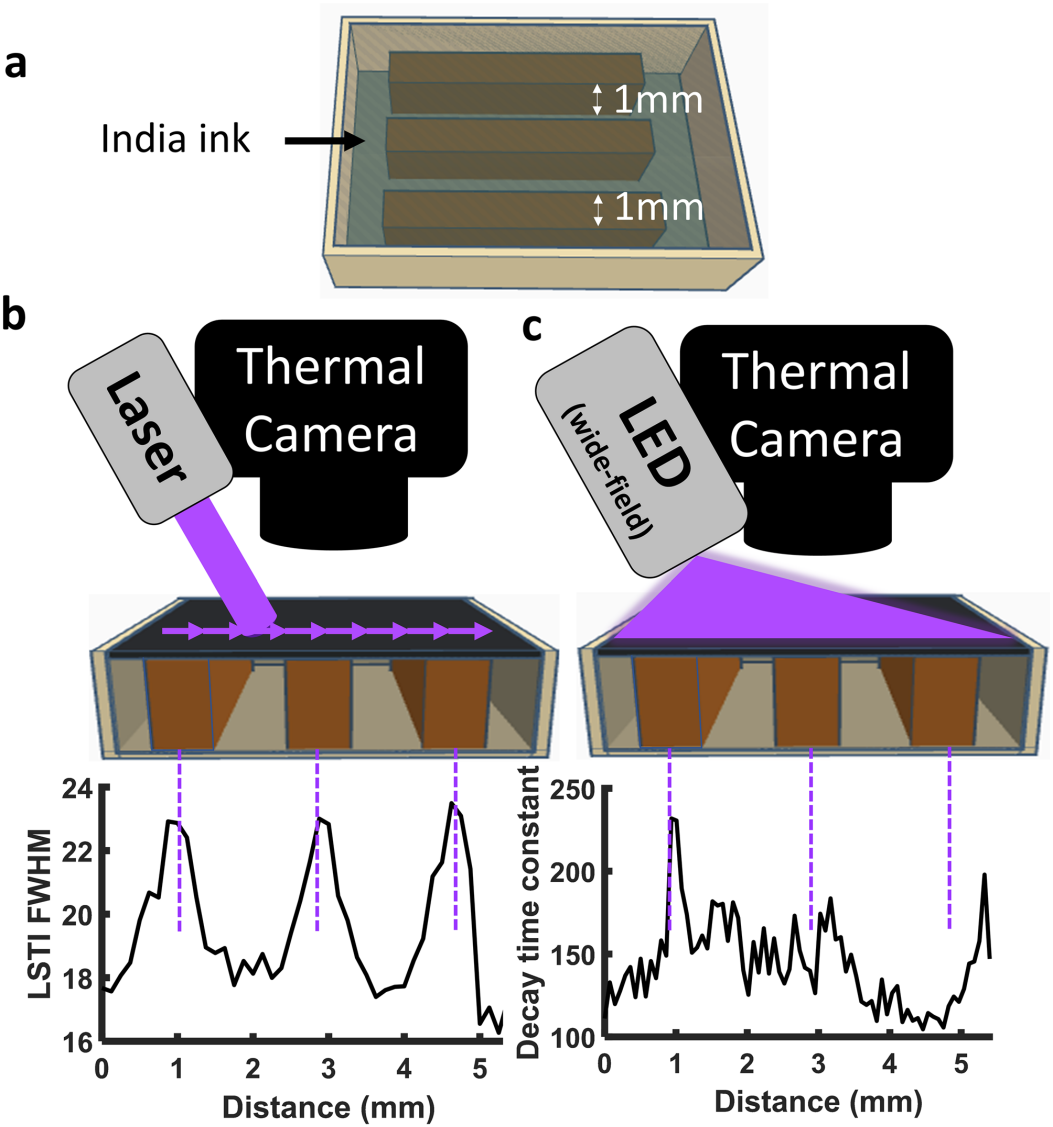
Testing spatial resolution with 3D printed phantoms (**a**) demonstrates superior spatial resolution for FDTI (**b**) relative to widefield dynamic thermal imaging (**c**).

The effective spatial resolution of FDTI depends on the spatial resolution of the thermal camera used, the laser beam width, and the step size between imaging points. A narrower laser beam generates heating over a smaller tissue volume than a wider laser beam, reducing thermal diffusivity-driven blur and high spatial resolution. The spatial resolution of FDTI and a standard widefield DTI using a 405 nm LED was tested using a custom 3D printed phantom that had 1 mm thick pillars printed in rows and spaced 1 mm apart (Fig. 3). The phantom was filled with India ink solution to obscure the pillars (Fig. 3a). FDTI measurements were taken in 125 micron steps across the phantom and the FWHM for each measurement was extracted and plotted for each step (Fig. 3b). The widefield LED illuminated thermal decay time constants were plotted pixel by pixel against the phantom location (Fig. 3c). FDTI shows three distinct peaks corresponding to the three pillars, whereas widefield DTI did not reveal structures consistent with phantom components.

### FDTI detects changes in tissue heterogeneity in vivo

Proliferating cells such as tumors can create a high level of disorganization and tissue heterogeneity by recruitment of blood vessels, de-differentiation of cancer cells into different phenotypes, and induction of a robust immune infiltrate. To determine if FDTI can measure malignancy-induced changes in tissue heterogeneity, subcutaneous mouse and rat models of breast cancer were evaluated over the course of tumor development. Two experimental designs with different healthy tissue control sites were investigated: one consisting of the selection of a contralateral site, and another consisting of a temperature-matched control site. FDTI images and videos were acquired in real-time with a thermal camera and a low powered (5 mW) laser-induced heat source. The thermal data were processed and analyzed to extract FDTI features based on tissue heat profile.

**Figure 4:**
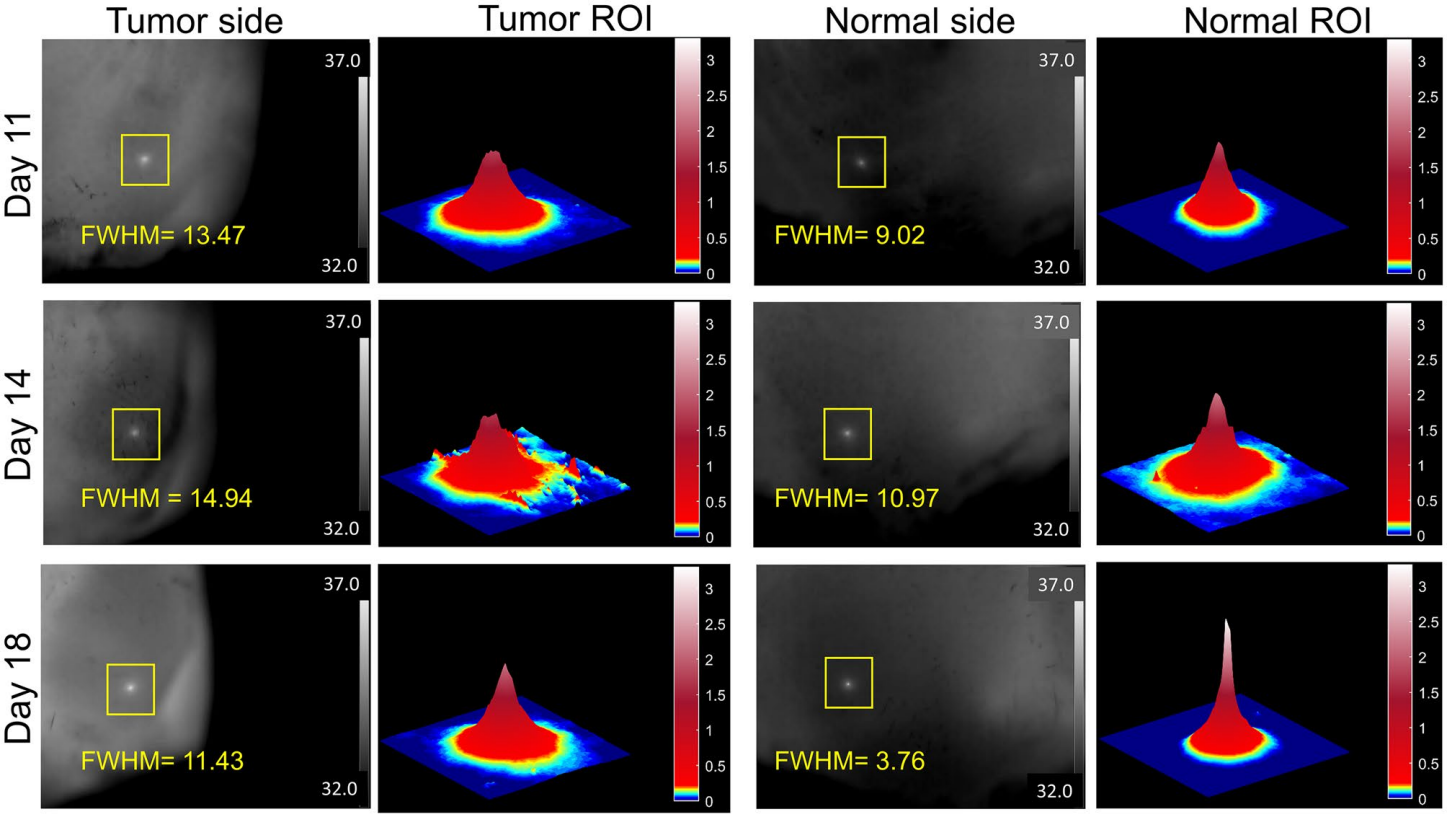
FDTI of one rat throughout tumor progression. Thermal image (left) and corresponding FWHM from FDTI analysis (left center) on tumor. Thermal image (right center) and corresponding FWHM from FDTI analysis (right) from normal tissue over time.

**Figure 5:**
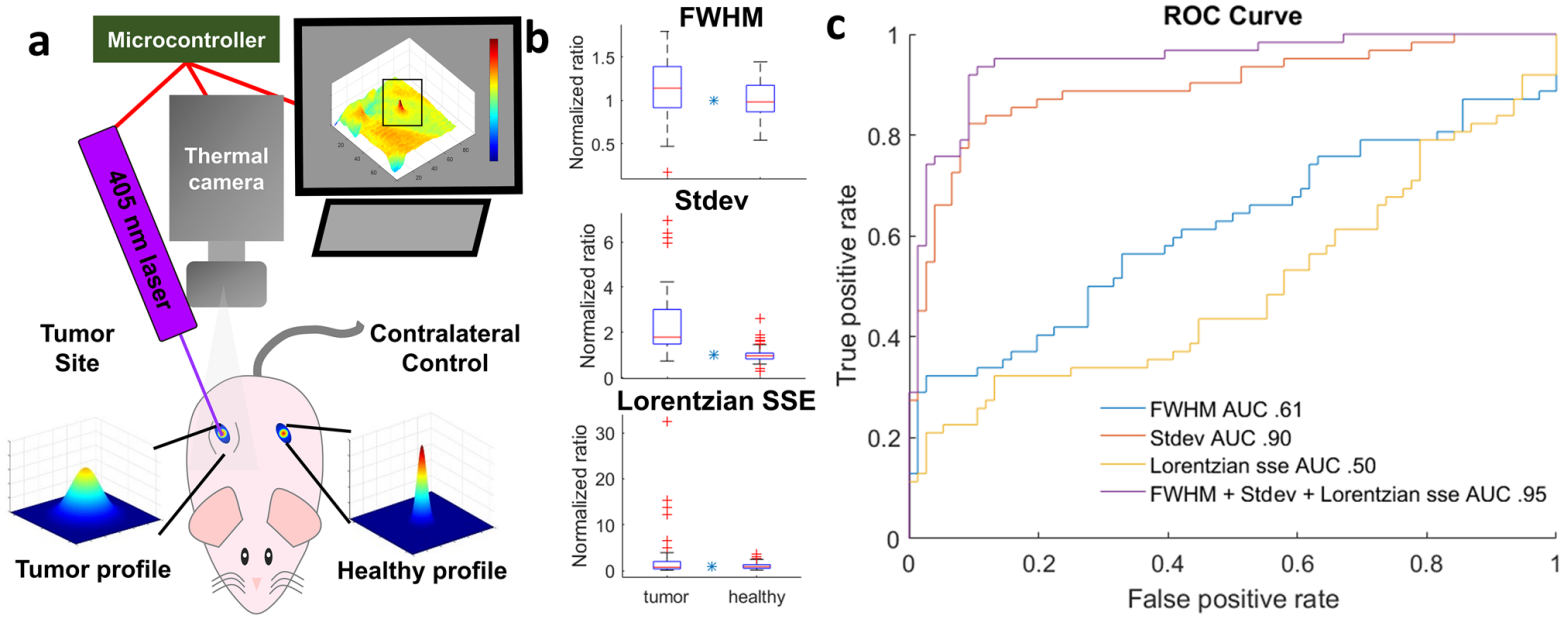
Demonstration of FDTI in the use of cancer detection in a mouse model of breast cancer. (**a**) FDTI experimental setup showing contralateral control experimental design; (**b**) FDTI feature responses between tumor and contralateral control healthy tissues in mice. (n = 9, *indicates *p* value < 0.05). (**c**) Receiver operator characteristic curves from fivefold cross-validation quadratic discriminant analysis in mice.

**Figure 6:**
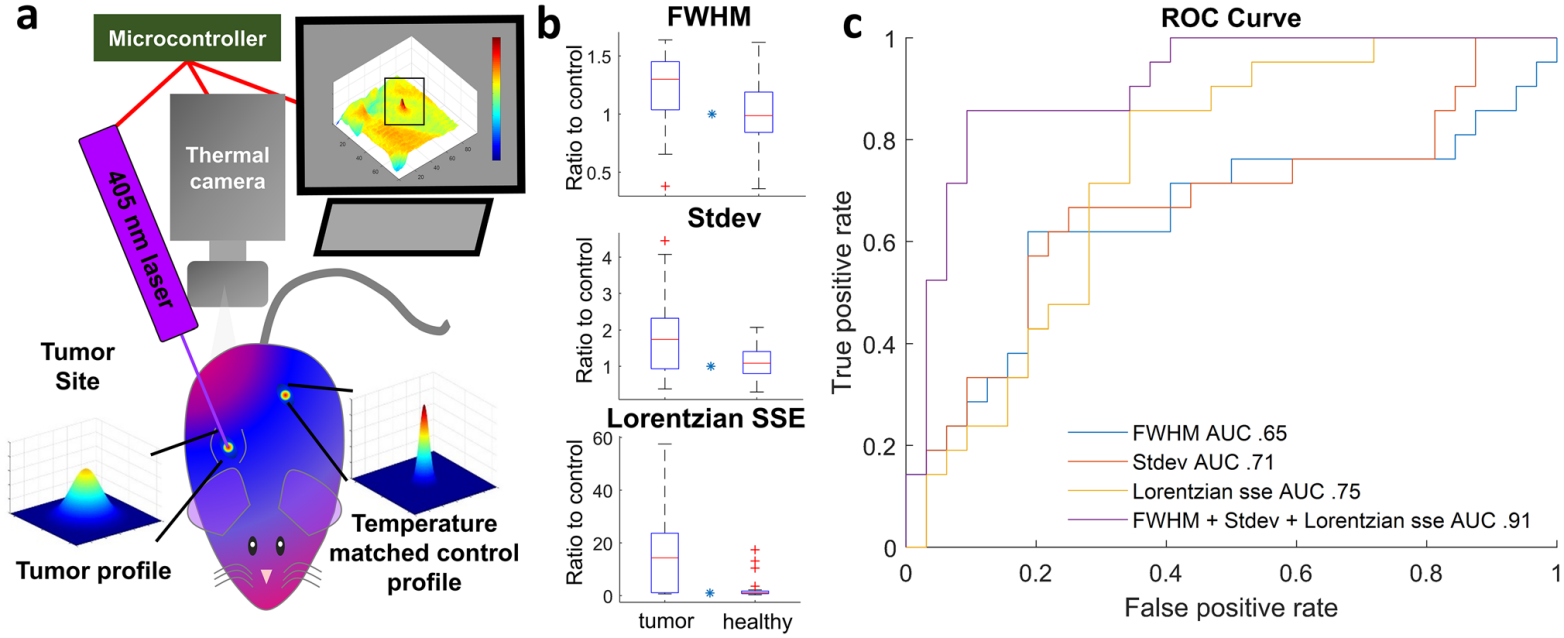
Demonstration of FDTI in the use of cancer detection in a rat model of breast cancer. (**a**) FDTI experimental setup showing and temperature-matched control experimental design; (**b**) FDTI feature responses between tumor and temperature-matched healthy tissues in rats. (n = 4, *indicates *p* value < 0.05); (**c**) Receiver operator characteristic curves from fivefold cross-validation quadratic discriminant analysis in rats.

A simplified visualization of the FDTI data was obtained by plotting the tissue thermal profiles after 10 s of continuous laser stimulation. This allows for a visual comparison of thermal profiles between tumor and healthy tissue. In the early stages, both tumor and healthy tissue exhibited similar but distinguishable heat profiles, measured by FWHM, but this feature diverged as the tumor size increased and the tumor matured (Fig. 4). In general, the healthy tissue exhibits a taller and narrower FDTI profile than tumor tissue, which showed a shorter and broader thermal response. As the tumor developed, the thermal response of healthy tissue became sharper and taller. Although the height of the thermal response in the tumor region increased slightly with time, the profile remained broad throughout tumor development. These results point to the potential use of high-resolution FDTI to determine tissue proliferation and heterogeneity. Furthermore, the visual features correspond to consistent findings observed across all of the mouse and rat measurements, which had significantly larger FWHM, standard deviation at steady state, and error when fitting the thermal profiles to a Lorentzian surface plot (Figs. 5 and 6).

### Multiparametric feature classifier enhances the accuracy of tumor detection in a mouse model of cancer

In the mouse experiment, Balb/c mice with subcutaneous breast tumors on the right dorsal flank (n = 9) had FDTI measurements made on the tumor and the contralateral sides which served as healthy control (Fig. 5a). Thermal images were collected 10 s after turning on the laser, features were extracted and compared with the contralateral-control group. Figure 5b displays results from three thermal profile features: FWHM, the Lorentzian fitting sum of squared errors (SSE), and the temperature standard deviation at steady state. These features are independent of each other and were tested as classification inputs.

Results from a fivefold cross-validation quadratic discriminant analysis are displayed in the form of receiver operator characteristic curves from the three features (Fig. 5c). The area under the curve (AUC) values of single features range from 0.49 to 0.89. However, when combined in a multiple feature classifier, the AUC reached 0.95, demonstrating higher accuracy than any individual feature.

### FDTI distinguishes underlying tissue heterogeneity in a rat model of cancer

Thermal imaging is widely used to map body temperature and identify suspicious areas, but the low specificity of the technique has hampered adoption in the clinic^4,14^. Multiple hot spots may indicate inflammation, infection, or cancer. A unique application of FDTI is the rapid assessment of suspicious lesions that are first flagged using the standard widefield thermal imaging systems. In such a case, it is more relevant to have temperature-matched comparison sites to determine whether thermal stimulation of a suspicious area of tissue can distinguish between cancerous and healthy tissue. In this study, Sprague Dawley rats with subcutaneous right flank breast tumors (n = 4) had three measurements acquired from the tumor, and three measurements acquired from temperature-matched sites (Fig. 6a). One of the temperature matched measurements served as the control, and all the remaining measurements were normalized to this control measurement. FDTI data were acquired in a video format, with recording initiated just prior to a 10-s laser exposure, followed by a 10-s observation of thermal recovery. FDTI features were extracted as described in the Methods section, and comparisons were made between features of the tumor and healthy group. Plots between healthy and tumor tissue from the same three features utilized for classification in the mouse study (FWHM, Lorentzian SSE, and steady state temperature standard deviation) were analyzed (Fig. 6b). The trends observed are the same, with tumor tissue having statistically significant higher values than the healthy tissue for all features.

Similar to the mouse study, these features were tested as classification inputs. A fivefold cross-validation quadratic discriminant analysis was displayed in the form of receiver operator characteristic curves from the three features above (Fig. 6c). The area under the curve (AUC) values of single features ranged from 0.65 to 0.75, but combining them into a multiple feature classifier improved the AUC to 0.91, demonstrating higher accuracy than any individual feature. In addition, the consistency in the multiple feature classifier between mouse and rat studies highlights the robustness of this technique and these features as predictive inputs.

## Discussion

**Figure 7:**
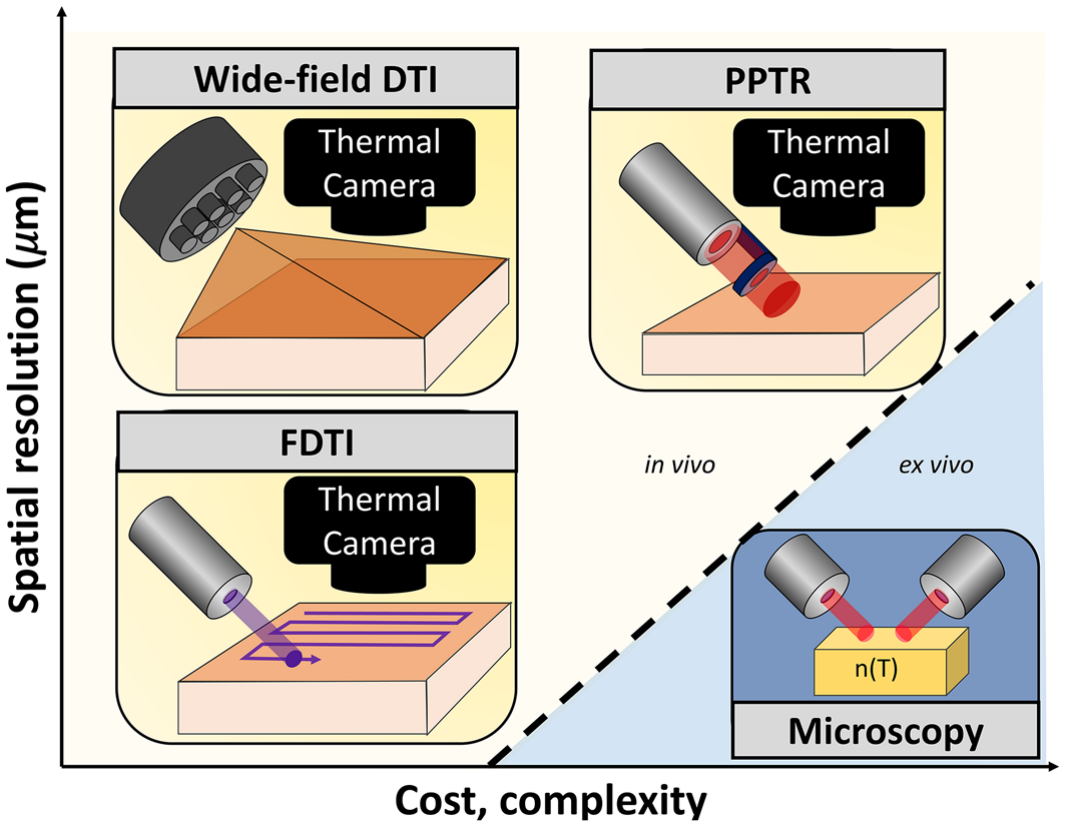
Comparison of dynamic thermal imaging techniques’ resolution and complexity.

Photothermal techniques, which measure the thermal response of a system to photon absorption, can be used to interrogate the thermal properties of materials. This includes measurement of multiple photothermal sequelae, including change in index of refraction, heating, thermoelastic expansion, secondary infrared irradiation, and photomechanical stresses^15^. For applications at nanoscale, scanning thermal microscopy techniques use thermal probes attached to cantilevers that directly contact the material to detect nanoscale changes in heat flow^16^, from which thermal properties can be extracted. At microscale, the 3ω method has been used to measure the thermal conductivity of materials and single cells^17,18^. A number of microscopes have been developed that take advantage of the local change in refractive index upon chromophore absorption, some of which use vibrational spectroscopy to extract molecular content^15,19,20^. Macroscopically, widefield thermal imaging is routinely used in industrial applications such as assessment of electrical connections and bulk material characterization, while DTI has been increasingly used in medical applications^21,22^. Few methods exist that can probe micro and mesoscopic-resolution applications without the use of bulky, expensive hardware, that limit field applications. To meet this need, we have developed FDTI (Fig. 7).

Existing photothermal methods have reported resolution at sub-micron levels^15,20,23^, however, the cost and complexity of these techniques have limited their use in the field. In medical applications, more translational, but significantly lower resolution techniques such as widefield DTI and pulsed photothermal radiometry have been studied. Pulsed photothermal radiometry (PPTR) is a non-contact optical method that applies a pulsed laser to generate heat due to optical absorption and measures thermal diffusivity based on heat propagation through the material^21^. This method has been recently used for physiologic characterization of skin^24^ and to predict temperature rise in patients undergoing laser therapy^25^. Both of these techniques apply stimulus over a large area and the subsequent thermal response becomes blurred due to thermal conductivity, significantly reducing the spatial resolution. A recent article achieved sub-diffraction limited thermal imaging of tissue slides stained with exogenous methylene blue nanoparticles for contrast enhancement by irradiating the sample using a scanning focal light beam to prevent thermal blurring^11^. This technique tracked optical absorption induced heating, but did not track heat dissipation over time, confining its sensitivity to optical absorption and specific heat capacity rather than thermal conductivity. Capitalizing upon the successes of these methods, FDTI irradiates small regions of tissue to prevent thermal blur and tracks the response over time, providing sensitivity to heterogeneity in optical and thermal properties. The sub-millimeter resolution demonstrated in our FDTI work is not the absolute resolution limit, rather the resolution is fundamentally limited by spot size, step size, and the thermal resolution of the camera used.

FDTI’s instrumentation is exceptionally simple, requiring only a collimated laser and a thermal camera. Furthermore, the extraction of FDTI features is straightforward and can be performed in real time. Indeed, the specifications of FDTI equipment will affect the resolution and sensitivity of the system, but these simple requirements are in stark contrast to high-resolution thermal techniques which utilize expensive laser systems^24^, lock-in detection schemes, fragile alignment systems^15,19–21,23^, or which rely on exogenous contrast^11^ which limits in vivo application. In addition, some of the lasers used are medical grade therapeutic lasers that cause pain^24^, which highly differ from the painless and low power irradiation used for FDTI.

FDTI was tested in an in vivo cancer detection application, a possible avenue to pursue in future human studies. A variety of widefield thermal imaging methods have been tested for cancer detection due to their favorable translational characteristics. Some methods aim to improve the accuracy of equilibrium thermal measurements using artificial neural networks and have reported accuracy of 80.95% in tumor detection^26^. DTI has been shown to increase the accuracy significantly (from 54 to 82%) when compared with steady state thermal imaging^27^. Improved classification algorithms have heightened the sensitivity and specificity of this technique to 95–100% and 83–99% respectively, for the diagnosis of skin cancer^5,28^ in a 140 patient trial. While these results are exciting, the spatial resolution limitations of widefield DTI techniques may not allow robust estimation of tumor heterogeneity and margin assessment, which would further expand the utility of dynamic thermal imaging. Additional heat challenge techniques have applied visible light sources including green LEDs that created heat due to absorption by hemoglobin to identify the vascular boundary of tumors^29^. Herein, FDTI had 95% and 91% accuracy in small cohorts of breast cancer mouse and rat models, respectively, using classification based on single point FDTI measurements of cold tumors. A number of quantitative image parameters indicative of cancer were identified from FDTI data. FWHM was significantly higher in cancerous regions, which have higher thermal conductivity than healthy tissues and can thus transfer heat away from the thermal source faster, preventing a large heat buildup. The standard deviation was higher in cancerous tissues and may be due to more heterogeneous tissue that can be observed in tumors. Finally, the goodness of fit to a Lorentzian surface as measured by the sum of squared errors was significantly lower in cancerous tissue, further suggesting that spatial inhomogeneities are more frequent in cancerous tissue and impact the likeness of the FDTI profile to a Lorentzian surface. Importantly, the quantitative nature of the FDTI parameters makes it an objective technique that should not be dependent upon the user or require interpretation, overcoming a large obstacle from standard thermography.

The simplicity of FDTI lends itself to being portable, fast, low cost, and lightweight, and is therefore prime for clinical translation and field applications. This is an important advantage compared to non-widefield thermal methods, which have thus far been performed on tissue sections or in vitro cell samples. Our successful range of experiments spanning 3D printed structures, ex vivo biological tissue, and longitudinal in vivo mouse and rat studies demonstrate the utility of this approach across many biomedical applications and size scales, and warrants further exploration of this technique in human patients. Widefield approaches which have similarly simple and portable instrumentation have shown great promise to positively impact patient care^5,22,28,30^, and FDTI is poised to fill the need for simple, inexpensive, and high resolution dynamic thermal imaging.

Development of real-time analysis software and further optimization of the laser irradiation and thermal decay times for scanning and extension into rapid 2D FDTI mapping could further improve FDTI. Although the classification accuracy of in vivo cancer detection was not perfect, the classification was made using single point FDTI measurement that cannot capture the full extent of the tumor’s heterogeneous properties. Repeating such experiments with 2D mapping capabilities in larger animal cohorts will be a fairer test of FDTI’s diagnostic abilities. Furthermore, depth penetration of the excitation light can be increased by using longer irradiation wavelengths^31^, and through longer laser exposure times^32^, however, deeper imaging will result in a trade-off of reduced spatial resolution^33^.

Interestingly, we observed that mouse and rat tumors progressively grew cold as they increased in size, which is contradictory to human tumors which are known to be warmer than surrounding tissue^34,35^. However, our finding of cold rodent tumors is supported in the literature^36,37^. This implies that most rodent models do not represent human disease with respect to an important thermal physiologic parameter. However, a strength of FDTI and dynamic thermal imaging approaches is the sensitivity to thermal properties of the tissue, regardless of baseline temperature. Temperature is known to affect immune response and enzyme activities^38^, and these important differences should be considered when conducting cancer research in animal models. Due to this difference in the tumor microenvironment, FDTI trials in humans may yield different results compared to those observed in cold mouse and rat tumors, and should be evaluated independently of the animal data presented herein.

In summary, we have developed FDTI, a platform technology that offers high-resolution and high contrast thermal imaging to applications with sample heterogeneity such as biological tissue. It adds measures of thermal properties to the sources of label-free optical contrast, with the potential to reveal new pathophysiology not yet before seen with conventional optical methods. FDTI addresses an important gap in stimulated thermal imaging with widefield DTI systems being inexpensive, simple, and clinic-ready but suffering from poor spatial resolution, and thermal microscopes offering high resolution but with costly, complex, non-portable, and ex vivo-only applications (Fig. 7).

## Materials and methods

### Experimental FDTI system

The instrumentation used to perform FDTI experiments is shown in Figs. 5a and 6a. The FDTI system consists of a collimated, low powered, continuous 405 nm laser diode (Laserland, Wuhan, Hubei Province, China). The laser beam profile was characterized and found to be an elliptical Gaussian with FWHM 0.69 mm × 1.35 mm. A polarizer is aligned with the laser beam to limit power output at 5 mW. A FLIR (Wilsonville, OR, USA) T650SC 25° high-resolution focal plane array uncooled microbolometer with 640 × 480 pixels, 64 mm × 48 mm field of view, 100 µm spatial resolution, < 0.02 °C sensitivity, spectral range of 7.5–14 µm, and 30 Hz frame rate is used with a 5.8 × Lens (FLIR #T198060) to collect thermal images and videos. The laser is powered using a custom electronic driver circuit controlled by a Teensy LC (Sherwood, Oregon, USA) microcontroller. Both the Teensy and camera are connected to a PC running a custom MATLAB script (MathWorks, Natick, MA) to allow for synchronized, automated control of each component. The script first triggers the camera to record thermal video prior to laser stimulation, then triggers the laser on for a specified pulse duration, and finally continues recording video after the laser is shut off for a sufficient time period to capture return to equilibrium temperature. The laser exposure time for in vivo subcutaneous tumor measurements was tested at 0.1, 1, 3, 5, 10, 20, 30, and 60 s to identify the shortest amount of time that reached a plateau in temperature rise, from which 10-s exposure was selected. Similarly, the time until thermal decay plateaued was observed and found to consistently decay within 10 s after laser exposure completion.

### Experimental image acquisition, processing, and feature extraction

Thermal images and videos are processed and analyzed using our custom image processing pipeline. First, thermal radiometric image or video data is imported into a custom MATLAB graphical user interface (GUI). The user is then prompted to select the laser irradiation point from a thermal preview image. A region of interest (ROI) encompassing the heated tissue is then programmatically generated. A battery of parameters, including peak temperature, thermal peak area, thermal peak volume, 2D Gaussian fitting, 2D Lorentzian fitting, and FWHM are subsequently measured from this ROI. In addition, measures of goodness of fit for temperature rise can be utilized in real-time to ensure appropriate experimental conditions.

### FDTI resolution testing

FDTI’s ability to resolve small spatial differences in material properties was tested using a 3D printed phantom composed of black polylactic acid (PLA) filament (Hatchbox 3D, Pomona, CA, USA) in which rows of 1 mm thick pillars were spaced 1 mm apart and filled with a 1000× dilution India ink solution (Higgins, Leeds, MA, USA). The India ink solution provides an optical absorber, and the water and plastic provide thermal conductivity differences on a small spatial scale. To test the spatial resolution as a function of laser beam width, the irradiation beam diameter was adjusted using a 1000 µm pinhole and the beam was translated across the pillars with a 125 µm step size. The data were analyzed by plotting the FWHM and the thermal decay constant was plotted as a function of translated distance.

The spatial resolution of FDTI was directly compared to widefield irradiation DTI techniques by irradiating the phantom with a 405 nm LED with a 1 cm spot size. As analyzed in the literature, the temperature decay after light exposure was fit to an exponential and the decay constant was calculated per pixel in the illumination area. A row spanning the same pillars measured using FDTI was analyzed and per pixel decay time constant was plotted to identify the spatial resolution and directly compare with the FDTI values from the same pillar spacing rows.

### Computational model

The FDTI phenomenon was modeled in COMSOL Multiphysics (Burlington, MA, USA) version 5.4.2 by coupling of the Bioheat Transfer and Radiation in Absorbing-Scattering Media modules. This software enabled simulation of heat transfer based on specific properties of the tissue using the finite element method on a custom mesh to solve for the change in heat over time. The model solved Pennes’ bioheat equation which requires input for the density, specific heat capacity, initial temperature, ambient temperature, and thermal conductivity of the tissue, blood perfusion rate, and metabolic heat generation, all of which were obtained from values published in the literature (Table 1). Laser-induced heating was incorporated into the model using the Radiation in a Participating Media module. The radiative heat term requires inputs of optical absorption coefficient, scattering coefficient, initial radiative intensity, refractive index, and coefficient of anisotropy. For boundary conditions, the surface was set as a diffuse surface with thermal emissivity and ambient temperature inputs as shown in Table 1. The interior boundaries are set to "open boundaries" and thus allow for heat to transfer through them, and the boundary temperatures are consistent with adjacent internal temperatures throughout the duration of the simulation. Prior to application of the radiative term, the tissue was allowed to self-regulate for 2 min, followed by the application of a 5 mW laser with a Gaussian profile on the tissue for 10 s.

**Table 1:** Parameters used in COMSOL parameter testing.

Parameter	Value(s) used	Sources
Absorption coefficient (1/m)	900 (baseline) 700 (low) 1075 (high)	^39^
Ambient temperature (K)	296.45	Room temperature
Initial tissue temperature (K)	303.45	
Tissue Density (kg/m^3^)	1000	^40,41^
Specific heat capacity of tissue [J/(kg K)]	3000	^41–44^
Metabolic heat (W/m^3^)	200	^45,46^
Thermal conductivity [W/(m K)]	0.35 (baseline) 0.21 (low) 0.48 (high)	^40,41,43,44,47^
Surface emissivity	0.98	^48^
Refractive index	1.40	^39^
Blood perfusion(1/s)	0.001	^49^
Scattering coefficient(1/m)	2500	^39^
Coefficient of anisotropy	0.90	^39^

### Computational parameter sweep

The effects of various model parameters on model output were tested using parameter comparison of tissue thermal conductivity and optical absorption at a range of values reported in the literature from physiological studies (Table 1). Parameter sweep results were analyzed by plotting the cross-section of the surface temperature. The amplitude and FWHM of the thermal peak generated were calculated throughout laser stimulation and compared across parameter values.

### Computational testing of biological tissue types

The ability to distinguish between tissue types was tested by evaluating the model using input parameters from fat and muscle tissue based on reported literature values (Table 2). Similar to the parameter sweep, the peak amplitude and FWHM were calculated throughout laser stimulation and compared.

**Table 2:** Parameters used in COMSOL validation of biological tissue types.

Parameter	Porcine fat	Source	Porcine muscle	Source
Absorption coefficient (m^-1^)	150 (λ = 405 nm)	^39,50^	950 (λ = 405 nm)	^51^
Ambient temperature (K)	296.45	Room temperature	296.45	Room temperature
Initial tissue temperature (K)	294.99	From experimental data	293.34	From experimental data
Tissue density (kg/m^3^)	911	^40,41^	1090	^40,41^
Specific heat capacity of tissue [J/(kg*K)]	2348	^41–44^	3421	^41,42,44^
Metabolic heat (W/m^3^)	0	Ex vivo, term ignored	0	Ex vivo, term ignored
Thermal conductivity [W/(m*K)]	0.24	^40,41,44^	0.56	^40,41,43,44,47^
Surface emissivity	0.98	^48^	0.98	^48^
Refractive index	1.40	^39^	1.40	^39^
Blood perfusion (s^-1^)	0	Ex vivo, term ignored	0	Ex vivo, term ignored
Scattering coefficient (m^-1^)	7750 (λ = 405 nm)	^51^	7000 (λ = 405 nm)	^51^
Legendre coefficient of anisotropy	0.90	^39^	0.90	^39^

An experiment was designed to test the accuracy of the COMSOL model. Bulk pieces of porcine muscle and porcine fat were experimentally measured using an FDTI system as described below. The amplitude and FWHM were calculated and plotted, and compared to COMSOL simulation results.

### In vivo mouse and rat experiments

All animal work was approved and performed under an approved protocol by Washington University’s Institutional Animal Care and Use Committee (IACUC). Animals were housed under a 12 h dark–light cycle.

Two subcutaneous breast cancer models were used. The first subcutaneous model utilized 6 week-old female Balb/c mice (n = 9) that were subcutaneously implanted with 10^6^ 4T1-Luc-GFP murine breast cancer cells on the right dorsal flank. The tumor size was tracked using calipers and were typically palpable by day 7. Images at steady state and at 10 s of laser stimulation on days 7, 8, 10, 11, 14, 16, and 18 were captured post injection. FDTI measurements were acquired on the tumor and matched contralateral sites served as controls.

The second subcutaneous breast cancer model used 6 week old and adult female Sprague Dawley rats (n = 2 each, n = 4 total), which were injected with 10^5^ MAT B III mammary adenocarcinoma cells in the right flank. FDTI measurements were acquired on the tumor and temperature-matched regions served as controls.

FDTI videos were taken of the rats every 3–4 days post tumor cell injection. Caliper measurements were taken to track the growth of the tumor. Once a palpable tumor was identified, points were measured on the tumor.

All animals were sacrificed based on humane end-points. Thermal images and videos were used for feature extraction using our image processing pipeline.

### Statistical analysis and classification

Statistical analysis was performed using MATLAB R2018a. Multiple image features were extracted from each FDTI image and video as described above. Each feature was correlated using the Spearman correlation against the class label (healthy or cancerous) and ranked to determine which features were most predictive of tissue composition. The ability to distinguish healthy from cancerous tissue based on the individual most predictive features was determined using a t-test with a level of significance set to 0.05. An additional correlation matrix comparing all features was calculated to identify independent features to serve as predictors. Individual predictors and combinations of them were entered to train and evaluate a Quadratic Discriminant Analysis classifier with fivefold cross-validation using the MATLAB Classification Learner Application. The model performance was evaluated using accuracy measured as area under the receiver operator characteristic (ROC) curve.

### Ethical approval

All methods were performed in accordance with the relevant guidelines and regulations (i.e. Declaration of Helsinki).

## Data Availability

The datasets generated and analyzed during the current study are available from the corresponding author on reasonable request.
